# Development and Structures of Trialogue for Bipolar Disorders in Germany and Guidelines of the German Society for Bipolar Disorders

**DOI:** 10.3390/medicina57111213

**Published:** 2021-11-05

**Authors:** Martin Schaefer, Marylou Selo, Nadja Stehlin, Barbara Wagenblast, Thomas Bock

**Affiliations:** 1 German Society for Bipolar Disorders e.V., 60528 Frankfurt am Main, Germany; mselo@aol.com (M.S.); n.stehlin@dgbs.de (N.S.); b.wagenblast@dgbs.de (B.W.); bock@uke.de (T.B.); 2 Clinic for Psychiatry, Psychotherapy, Psychosomatics and Addiction Medicine, Evang, Kliniken-Essen-Mitte, Henricistrasse 92, 45136 Essen, Germany; 3 Department of Psychiatry and Psychotherapy, University Medical Center Hamburg Eppendorf (UKE), 20251 Hamburg, Germany

**Keywords:** bipolar disorders, German Society for Bipolar Disorders (DGBS), participation, peer support, trialogue

## Abstract

The German concept of a trialogue in medicine is at its best a cooperation between patients, relatives, and professionals as partners on equal footing. Prerequisites, and also the aim of the trialogue, are mutual respect, an open attitude from professionals, and self-confidence from patients and relatives. The expertise of each of these groups is to be strengthened through the trialogue and should benefit all. Trialogue cooperation brings about a change of perspective and promotes mutual understanding. By establishing a therapeutic relationship on equal footing with the patient with involvement of their relatives, individual and family resources can be better utilized, professional assistance can be designed to better meet the patient’s needs, and acceptance of and commitment to treatment can be increased. In addition, early symptoms and new phases of the disease can be recognized earlier and adequate treatment can be initiated more quickly. A favorable course of the disease is thus more likely, and relapses are less likely to present. The use of peers has proven to be quite helpful. The consistently trialogue structure within the German Society for Bipolar Disorder (Deutsche Gesellschaft für Bipolare Störungen e.V./DGBS: Heinrich-Hoffmann-Straße 10, 60528 Frankfurt am Main) as a medical society enables further development of the trialogue on many levels, for example, the drafting and updating of the German guidelines for bipolar disorder with the trialogue in mind.

## 1. Introduction

In Germany, the idea of a “trialogue” in psychiatry arose at the end of the 1980s from a meeting between Dorothea Buck and the clinical psychologist Thomas Bock, where it was jointly decided to promote the exchange between professionals and patients and also to include relatives in the therapeutic process. Dorothea Buck had experienced the often deadly and always silent national socialistic (Nazi) psychiatry herself and dedicated her life to teaching psychiatry, according to the motto: “If we talk with each other, at least we won’t kill each other”. Thomas Bock recognized the opportunity to develop a more open understanding of mental illness and a different mode of interacting between patients, relatives, and professionals. An important principle between patients (as experts of their illness), relatives, and specialists is to understand that mental illnesses are not exclusively pathological [1]. The trialogue arose in psychosis seminars, in which psychosis was phenomenologically defined as changes in perception, thinking, and affect. From the beginning, people with schizophrenic and bipolar affective psychosis were involved [2]. The focus is on mutual learning, a common language, an exchange of subjective experiences, and the opportunities for a change of perspective. For patients, these narrative processes have their own therapeutic effect (“psychotherapy without intention”). The most important effects are that patients and relatives learn from each other, precisely because they come from the same family, and professionals can reflect on their experiences in direct exchange because they do not have a therapeutic mandate. Thus, the risk of self-stigmatization can be reduced, and the social perception of all groups can be improved. In particular, the development of a “nonviolent discourse” (Habermas) is important against the background of German history (see above).

By reducing existing mutual prejudices between experts by experience, relatives, and professionals, it was possible to jointly fight against public and psychiatric prejudices. Different anti-stigma projects based on the trialogue or grass-roots initiatives (e.g., “Irre menschlich”, “Hamburg/Leipzig”) strive for more sensitivity and tolerance towards patients and others [3]. The diverse opportunities to meet face to face with the public in schools, churches, companies, through trialogue training for teachers, youth welfare, police, clergy, journalists, etc., presents a challenge for local politics. The anthropological understanding developed here is a prerequisite for any anti-stigma work and also improves the chances of reappropriating experience and thus psychotherapy [4,5].

The first world congress on the trialogue was held in 1994. This was the first on German soil after the Nazi crimes against the mentally ill. This critical discussion on “megalomania” and “linguistic confusion” in psychiatry under the motto “Farewell to Babylon—Understanding Boundaries in Psychiatry” broached the issue. Since then, more than 100 psychosis seminars or trialogue forums have been held on other phenomena/disorders, as well as trialogue projects on many levels, such as treatment agreements, complaints offices, advanced education, apprenticeships, and also in research (e.g., EmPeeRie Hamburg). The next important step was that participation and the trialogue became part of the German treatment guidelines for bipolar disorders from the year 2012 [6].

The concept of “open dialogue” originated in Scandinavia. According to this method, initial contacts in acute psychoses preferably take place on site and involve all parties. Here, the goal is to keep the psychiatric definition low-key/modest and to better understand the current resources, contradictions, and conflicts as a result. Through trialogue-designed learning processes, new forms of treatment may be cautiously tested and developed into structures that better enable the participation of patients and relatives in making important decisions. These include, most importantly, home treatment and peer support for experts by experience [7] and relatives [8] or projects that combine both. Outreach services in particular might need a trialogue character, an open understanding of mental disorders, an appropriate culture of encounter, and an awareness of family and social resources.

## 2. Types of Trialogue

A trialogue can exist on various levels and always promotes mutual respect, a common language, and the ability to change perspectives. This is important on different levels—in psychosis seminars or trialogue forums as a nucleus, in everyday psychiatric practice and therapeutic processes, and at the level of psychiatric policy. Direct trialogue can take place in family discussions, in groups for relatives, and in psychoeducational or self-help groups [9]. The more extensive political trialogue can be expressed in anti-stigma work, in efforts for sensitivity and tolerance, in participatory research, in complaints offices, or in visiting commissions (according to local psychiatry laws). Several organizations in Germany in this field are now trialogue-based, including the “Aktionsbündnis Seelische Gesundheit” (Action Alliance for Mental Health), the Trialogue Forum of the German Psychiatric Association (DGPPN) as well as regional anti-stigma or research projects such as “Irre menschlich” (https://www.irremenschlich.de date of access 11 May 2021 (© 2021–2021, Irre menschlich Hamburg e.V.)) or EmPeeRie (Empower Peers to Research). A structure that has been particularly well-developed over the past 20 years exists within the German Society for Bipolar Disorders (DGBS, Deutsche Gesellschaft für Bipolare Störungen e.V.), which is organized on the basis of the trialogue as a medical-scientific professional society. It is involved in self-help, counseling and education, nurturing the trialogue, and destigmatization. The DGBS is also involved in scientific projects, as well as in the drawing-up and updating of the German guidelines on bipolar disorders [10]. Moreover, the DGBS Board is composed on the basis of trialogue (https://dgbs.de date of access 11 May 2021).

In recent years, there have been increasing attempts to incorporate trialogue elements into the clinical treatment and communication process. This aims to improve the situation for patients, relatives, and professionals, especially in acute treatment phases, but also for the development of long-term strategies. In this context, participation (involvement of patients and relatives in the clinical decision-making processes and therapy goals), establishing treatment agreements, peer support, office hours for relatives, increased joint and subject-oriented information, and psycho-education offers are important [11]. “Therapeutic trialogue” is the mutual understanding and—as far as possible—cooperation on equal footing between patients, relatives, and specialists in the context of the respective psychiatric treatment situation. The prerequisite and simultaneous goal is to achieve an open attitude of professionals as well as informed and self-confident patients who are increasingly seen as “experts in their own cause”. The perception that professionals regard them as experts in their own field already has a positive effect on some patients and their relatives (empowerment). Relatives can make themselves heard and thus provide important information on the family background, the course of the disease, and onset of symptoms. They can also play an important role in coping with everyday life, providing support, and preventing relapses. Finally, the trialogue-oriented therapeutic work enables a change of perspective. It can strengthen the willingness of all participants (including of the professionals) to work together in a long-term, flexible, and respectful manner. Thus, a favorable course of the disease becomes more likely, and relapses become less frequent.

## 3. Trialogue Guidelines and their Significance for Therapeutic Trialogue

The German Society for Bipolar Disorders (DGBS), as a medical-scientific professional society based on trialogue, has developed guidelines for diagnosis and treatment of bipolar disorders, in which one chapter deals exclusively with the evidence and importance of trialogue [9,11]. Trialogue was early on firmly integrated in the shaping of this society and is considered extremely important for knowledge transfer and self-management, especially the boosting and strengthening of existing social skills [6]. Below, the guideline recommendations, in as far as they are relevant for therapeutic trialogue, will be explained. Due to the lack of prospective randomized studies in this field, the recommendation grades are almost exclusively “statements” or “clinical consensus points (CCPs)”. All recommendations were subjected to an intensive trialogue discussion during their development and were accordingly coordinated at “eye level”.

### Guideline Recommendations on Trialogue in Germany

The trialogue-specific recommendations from the updated guidelines for diagnosis and treatment of bipolar disorders [11] are listed in Table 1. Professionals should increasingly become aware that it is helpful and ultimately even necessary to speak not about but with the patients and relatives, to take them seriously as experts by experience on the basis of their own illness, and to respect them as equal partners. In the context of bipolar disorders, a trialogue with the natural inclusion of relatives has a special justification, since the relatives themselves are strongly involved and burdened by the range of phases and the extreme mood swings. Relatives should be involved as early as possible, provided that the patient agrees. Even if the patients often refuse to involve their relatives in the acute phases of the illness, therapists should obtain their consent for trialogue discussions after the symptoms have subsided. Sometimes, however, the relatives themselves seek help or are the first to pave the way to professional help. Creativity and flexibility are important here. It should be noted, however, that clinicians must always adhere to the patient’s will and protect the patient’s data unless there is an imminent threat.

A trialogue promotes mutual understanding and acceptance of necessary therapeutic measures. On the part of patients, a trialogue leads to greater acceptance of responsibility, more active self-determination, and improved self-management skills. Relatives can benefit from a trialogue because it can strengthen acceptance and self-protection and reduce the individual burden. Professionals benefit through an increase in empathy, relationship skills, flexibility, and continuity. Thus, the trialogue should be integrated into the training of professionals. Attending trialogue forums can help professionals to develop a different understanding of the disorder.

Table 2 includes recommendations for participatory decision-making in the form of CCPs (clinical consensus points, expert level). Based on equal communication, the model of “participatory decision making” refers to shared decision-making between those providing treatment and those affected by the illness [12,13,14]. It is intended to replace the paternalistic view, in which the physician alone knows what is good for the person concerned. However, this requires more time and transparency on the part of professionals, as well as an understanding on the part of those affected that they are dependent on help and a willingness to engage in critical self-observation and trust. If the personal situation of the patient and his or her relatives permits, and if the patient and his or her relatives agree, the relatives should also be involved in deciding on the desired treatment concept and the treatment goals. Jointly developed treatment agreements for emergencies facilitate decisions in crisis situations and can help avoid coercion. So far, only a few clinics in Germany routinely use such agreements, despite good empirical data.

The therapeutic concept of a trialogue also includes the mutual transfer of knowledge through various channels [15,16]. The recommendations for this are presented in Table 3. Patients and their relatives have a particularly high need for information at the onset of bipolar disorder or at the time of diagnosis. Professionals should take the time to give advice on the various information and support options. They should always take individual and social characteristics into consideration. Patients and their relatives need clear and comprehensive information about bipolar disorder in general, about the possibilities, side effects and risks of therapy, and about rehabilitation options. This should be done in a non-patronizing manner using a language as free of stigma as possible.

Therapies for patients should be offered in group settings whenever possible; this applies to psycho-therapeutic, peer-supported recovery, and self-help groups. A great advantage of group settings is that the exchange with others can counteract the illness-related fixation on the current phase and creates a tendency toward centering. Such groups can be easily integrated into the daily routine of a clinic or as an outpatient offer; ideally, they serve as a bridge between the outpatient clinic and the ward and can be used across settings.

Self-help groups are another important component in the area of bipolar affective disorders. They can be built on the trialogue and/or contain trialogue elements. They take into account existing social skills and the particular importance of self-esteem. They provide individual information about specific local therapy options and can provide an overview of the various manifestations and effects of bipolar disorder. Family groups help participants to become aware of their own stress. Participants can find the appropriate behavioral strategies for themselves from the coping strategies that have been presented. The confidential character of group meetings also strengthens self-reflection. The eternal balancing act between giving too much help, exercising too much control, and being patronizing on the one side and offering too little support on the other remains a learning process for all relatives.

Patient and family advocates not only provide information and education, but they can provide emotional relief for patients and their families. They also make suggestions for improving communication between relatives and patients. Self-help manuals go beyond the objectives of patient guidebooks and aim to instruct patients and their relatives in the independent implementation of therapeutic procedures and techniques (self-management). Counseling offers by e-mail or by telephone provide information on request anonymously, have a low threshold, and are personal and individual.

Self-help encompasses all assistance that patients and their relatives experience outside the professional help system. A distinction should be made between individual self-management through training programs, attending self-help groups (for patients and/or relatives in which primarily the symptoms of the disease, questions and problems relating to dealing with the disease, and its treatment options are discussed), and self-help forums on the Internet, which are accessible around the clock and in rural areas sometimes represent the only opportunity to exchange experiences (e.g., www.bipolar-forum.de) or similar groups in social networks.

Other important components are peer support and family support, as listed in the recommendation from the German guidelines in Table 4, respectively. Peer support is based on experienced patients who have received qualified training for this purpose (e.g., EX-IN training to become recovery facilitators) being involved in the treatment and care of people in mental crises. Peer support and the term EX-IN stands for a model launched by the European Union in 2005. The model is based on the belief that people who have gone through psychological crises can use these personal experiences to understand and support other people in similar situations. Peers often find it easier to gain the trust of patients than professional helpers, especially in times of crisis, when peers are particularly well placed to put themselves in the shoes of the patients because of their own experience (“we have been there, we can help”). Peer support has been scientifically shown to improve the quality of life of patients and increase treatment success, which in this case led to a recommendation grade B [8,17,18,19,20,21]. One of the special services of “peer experts” is to be able to get in contact with hard-to-reach patients [22] and be able to act as translators, advocates, bearers of hope, and mediators, as well as pillars of support during treatment (see also www.ex-in-deutschland.de). Especially for patients with bipolar disorder, peer-supported services have a high value. The latter are often lower threshold and easier to accept, and can bolster stigma resistance and self-efficacy.

The unpredictability of the mood swings in bipolar disorder places a great burden on family members. Some relatives are continuously burdened during acute phases of the illness, and, over time, their own health stability becomes compromised. Many partnerships cannot withstand the strain and many families break up. Children in particular are exposed to a wide range of stresses—with a high risk of falling ill themselves. At the same time, however, the involvement of the family in the treatment of bipolar patients often improves the course of the illness and reintegration into “normal” life. Therefore, the treatment of bipolar patients should not be carried out without family support, i.e., the appropriate consideration of the family context and without the involvement of relatives. The involvement of relatives can take place in the regular treatment setting, in particular by family discussions with patients (e.g., in the “Open Dialogue”), psycho-educative groups of relatives, or in family self-help groups. Other information options include family information days, family consultation hours, a counseling hotline, and family associations. The goal is to get relatives to provide information about symptoms, background, and the course of the disorder from their point of view; they are encouraged to report on their observations of the effect and side effects of medications, and to provide emotional relief for other relatives. They are given appreciation for their assistance to patients, coming up with help options for relatives, easing of illness-related conflicts between patients and relatives, and relapse prevention.

## 4. Therapeutic Trialogue in Psychiatric Treatment: Are There Limits?

It is undisputed that patients and their relatives should be involved in all stages of planning, offering, and evaluating psychiatric services. However, views differ on the extent to which they should be on an equal footing, having truly “equal participation” [23]. In psychiatry, there is a higher degree of uncertainty for historical reasons, but also because of the lack of objective diagnostic criteria. It is much harder to convince psychiatric patients and their relatives of diagnostic assessments and treatment approaches than for physical illnesses. Thus, to the extent possible, relatives should be informed about the processes for diagnostic decisions and therapeutic planning. A lack of insight into the illness or the rejection of the current treatment concepts make a therapeutic trialogue approach at eye level more difficult, and at the same time, more necessary than ever. The attempt by physicians to—in a trialogue sense—get back on eye level with patients by renouncing their own fixed orthodox medical views represents an opportunity. Perhaps by accepting the interpretation and views of the patients, it becomes possible to at least come up with a means of helping or even finding a treatment consensus without insisting on the existing classical diagnosis system and understanding of the disease. Even in a well-functioning trialogue, patients and their relatives must bear in mind that “having an opinion” does not qualify them to have a comprehensive say in making a diagnosis or considering a therapy or interpreting the results of research and science. This implies that the evolved trialogue needs rules and time for increased learning and development of the desired common level (“eye level”) [24].

Furthermore, a trialogue does not imply that roles can be exchanged. The patient—as expert in his or her own case—is not a trained physician, the well-read family member is not a scientist, and the physician alone cannot assess all the problematic needs of the patient and those of his family. However, psychiatry-experienced persons as experts in their own case (e.g., EX-IN recovery companions) are on the way to take on important functions in counseling, everyday support, and co-therapeutic tasks.

Trialogue work in therapeutic everyday life has to be learned. Trialogue in psychosis seminars or trialogue forums is helpful. However, it cannot be equated with therapeutic trialogue in everyday life, since completely different rules apply, and the meetings should take place on neutral ground without therapeutic dependence. Thus, it would be useful if participation in psychosis seminars or trialogue forums were a fixed component of medical-therapeutic training.

## 5. Limitations

Although data on trialogue for bipolar diseases were evaluated for the German Guidelines for Bipolar Disorders, only expert opinions or statements could be formulated, since in most areas there was insufficient evidence for recommendations. Due to the lack of scientific studies and scientific evidence, the report could not follow the scientific rules for systematic reviews.

## 6. Conclusions

German psychiatry played a dramatic role during the Nazi era. Many German psychiatrists actively contributed to the forced sterilization or collective murders of psychiatric patients. Until the 1970s, the chronically mentally ill and the mentally handicapped were neglected and marginalized from society. In addition, the patriarchal composition of medicine required a comprehensive reform of psychiatry. It became increasingly important to initiate a change in the way doctors, patients, and their relatives interact. The development of psychosis seminars enabled professionals and patients to have an open exchange for the first time, allowing them to learn from one another. Thus, it was possible to largely dispense with the hierarchical relation between them. The time had come to respect patient’s rights, have patients and doctors learn from one another, and accept the active involvement of patients and relatives in the medical decision-making process, especially in psychiatry. In Germany, the concept of a trialogue was encompassed in the German Society of Bipolar Disorders (DGBS e.V.) in 2000 and continuously furthered over the past 20 years. While the word “trialogue” is unknown and not used in many countries, the concept of a trialogue is being applied in practice in many places around the world today. In psychiatry, a trialogue takes for granted the desire to actively create an encounter “on equal footing” of people with mental diseases, their relatives, and professionals in the mental health field. At the same time, a trialogue is a good way to promote this goal. In “psychosis seminars” or “trialogue forums”, the effort to achieve a common exchange takes place on neutral ground and, as a rule, is independent of therapeutic dependency or family entanglement. In everyday clinical practice, a trialogue aims at developing joint strategies for the acute and long-term treatment of bipolar disorder involving patients and their relatives. Psycho-educational elements can also be useful in this process if they take into account subject-oriented, individual, and social characteristics and strive for participative decision-making. The use of peers in psycho-education and exchange of information, but especially in relationship-building, long-term support, and outreach assistance, is considered as particularly authentic and effective. Self-help groups for patients and relatives are another important pillar in dealing with Bipolar Affective Disorder and should be actively encouraged.

At present, implementing a trialogue action is not always easy; however, it is particularly important when dealing with bipolar patients. Trialogues represent a very important path to treatment success, because they fully take into account the social skills and specific self-esteem problems of patients. A trialogue presents an opportunity to achieve a significantly improved therapeutic relationship. Therefore, treatment success is usually considerably more likely, and dealing with the disease will become easier for both patients and their relatives. With the German Society for Bipolar Disorders as a pioneer of the trialogue movement, the trialogue has become an integral part of treating bipolar disorders in Germany in a well-established and overall respected way. Since the DGBS is a recognized medical-scientific specialist society, jointly agreed S3 guidelines have received a high level of acceptance and represent the highest possible therapeutic standard recognized by all.

## Figures and Tables

**Table 1:** Guideline statements regarding trialogue between patients, relatives, and professionals.

Trialogue for Patients, Relatives, and Professionals (Statements)
Special attention should be given to the trialogue aspect in professional training and advanced education. The direct participation of engaged patients, relatives, and other caregivers should be a matter of course.
In the context of treating bipolar disorder, trialogue cooperation is particularly important. It is an essential prerequisite for open, trusting, and successful cooperation between patients, relatives, and other caregivers as well as treatment providers, on the basis of which common interests and treatment goals can be pursued. Results of trialogue cooperation are not limited to the individual therapeutic relationship, but have an impact on the appropriate representation of the interests of patients and relatives in public and politics, on the promotion of quality and on the further development of care structures. Participation in trialogue forums can benefit disease management.

**Table 2:** Recommendations for Participatory Decision Making (PDM).

Recommendations for Participatory Decision Making (CCP *)
Beyond the legally prescribed duty to inform the patient, patients should be involved in the decision-making process regarding treatment strategies and desired effect. Possible risks and side effects also need to be addressed. This participative decision-making process is to be made with the practitioner, patient, and, if agreed, also relatives. The fact that the patient is well informed is the basis for cooperative decision-making and a prerequisite for health-promoting behavior. People with insufficient knowledge of German should be able to receive this information in their native language.
Written treatment agreements can help prevent critical phases and reduce the risk of coercion. Whether this is successful strongly depends on the quality of the agreement and the communication process.

* Clinical consensus point.

**Table 3:** Knowledge Transfer in Trialogue Work, Self-Management, and Self-Help.

Knowledge Transfer in Trialogue Work, Self-Management, and Self-Help
Appropriate information transfer influences the willingness of patients to cooperate and adhere to treatment; it also impacts self-confidence and quality of life. A friendly way of interacting with one another is crucial. (Statement)
Patients and relatives should be made aware of available support systems, be it counselors, self-help manuals, training programs (e.g., communication training, self-management training), specific literature references, and should be encouraged to participate in current activities. (CCP *)
Counselors and self-help manuals should be independent of commercial interests, easy to understand, and of high quality. They do not replace psycho-education, but supplement it quite well. (Statement)
Self-management should be continuously bolstered in the therapeutic process. In this process, peer support can effectively complement self-help. (CCP *)
Patients and their relatives as well as other caregivers should be encouraged to attend self-help groups. The concrete naming of the (nearest) contact points (e.g., NAKOS **, DGBS, other associations of relatives) is helpful. Self-help groups should receive more attention as a therapeutic option. In addition to direct integration into in-patient therapeutic services, continuous cooperation with regional groups or a contact point for self-help groups is also conceivable. In this way, self-help groups can be used as an element of aftercare to stabilize the success of treatment. (CCP *)
Self-help groups should be supported by professionals by: • tangible encouragement to patients and their relatives to attend self-help groups • providing rooms in social institutions, churches, psychiatric clinics/offices • promoting offers of local self-help in notices, flyers, posters, etc. in social institutions, churches, psychiatric practices, hospitals, and offices • consciously designed transitions from professional to self-help groups • offers of ongoing counseling and support in crises (CCP *)

* Clinical consensus point; ** National contact and information point for stimulating and supporting self-help groups (Berlin, Germany).

**Table 4:** Peer Support and Family Support.

Peer Support and Family Support
Bipolar patients should be offered peer support to promote self-efficacy, self-management, adherence, or participation. (Recommendation grade B)
Family members should also be encouraged to provide peer support to reduce their burden and improve their quality of life. (CCP *)
Relatives should be involved from the beginning and throughout all phases of the treatment of the patient. (CCP *)
If the patient or family member refuses to be involved, efforts should be made to strengthen the relationship of trust between the patient and family member in the interest of ensuring long-term treatment success. (CCP *)

* Clinical consensus point.

## Data Availability

Not applicable.
